# Uterine cesarean scar resection during repeat cesarean delivery to prevent uterine niche formation: a randomized controlled trial

**DOI:** 10.1186/s12884-026-09142-w

**Published:** 2026-05-04

**Authors:** Ahmed Alnezamy, Ahmed Zaineldin, Ahmed Ghareib, Waleed Tawfik, Ahmed Sabra, Shreen Aboelezz

**Affiliations:** Department of Obstetrics and Gynecology, Faculty of Medicine, Benha University, Benha, Egypt

**Keywords:** Repeated cesarean section, Cesarean section scar defect, Uterine niche, Scar resection, Residual myometrial thickness, Randomized controlled trial.

## Abstract

**Background:**

A uterine niche (cesarean section scar defect), defined as an indentation ≥ 2 mm in the myometrium at the site of a previous cesarean scar, reflects impaired myometrial healing after cesarean delivery. Its prevalence increases with the number of cesarean deliveries and is associated with abnormal uterine bleeding, pelvic pain, secondary infertility, cesarean scar ectopic pregnancy, placenta accreta spectrum disorders, and uterine rupture in subsequent pregnancies. While secondary surgical correction may alleviate symptoms, preventive strategies implemented at the time of repeat cesarean delivery remain insufficiently studied. This trial evaluated whether resection of the previous cesarean scar during repeat cesarean delivery reduces uterine niche formation and related morbidity without increasing operative risk.

**Methods:**

This single-center, prospective, parallel-group, randomized controlled superiority trial was conducted at a tertiary university hospital between February and November 2025. A total of 170 multigravida women aged 18–45 years with ≥ 1 prior cesarean delivery undergoing elective or urgent repeat cesarean section were randomized (1:1) to either repeat cesarean delivery with resection of the previous cesarean scar (scar-resection group, *n* = 85) or standard repeat cesarean section without scar resection (control group, *n* = 85). Outcome assessors were blinded to group allocation. The primary outcome was the incidence of uterine niche (≥ 2 mm) at 6 months postpartum, assessed using saline-infusion sonohysterography. Secondary outcomes included operative time, additional hemostatic sutures, estimated blood loss, niche dimensions, residual myometrial thickness, and cyclic menstrual bleeding abnormalities at 6 months postpartum. Analyses were conducted on a modified intention-to-treat basis excluding participants lost before outcome assessment.

**Results:**

A total of 160 participants (80 per group) completed the 6-month assessment. Operative outcomes, including total operative time, number of additional hemostatic sutures, and estimated intraoperative blood loss were comparable between groups (all *P* > 0.05). At 6 months postpartum, the incidence of uterine niche was significantly lower in the scar-resection group than in the control group (45.0% vs. 78.8%; relative risk 0.57, 95% CI 0.44–0.75; *P* < 0.001), corresponding to an absolute risk reduction of 33.8% and a number needed to treat of 3. These results remained significant after adjustment for number of previous cesarean deliveries. Among women who developed a niche, scar resection was associated with significantly smaller niche depth, length, and width, greater residual myometrial thickness, and significantly fewer postmenstrual or intermenstrual spotting days and total bleeding days per cycle (all *P* < 0.0001).

**Conclusions:**

Resection of the previous cesarean scar during repeat cesarean delivery significantly reduces the incidence and severity of uterine niche formation, improves residual myometrial thickness, and decreases niche-related menstrual morbidity, without increasing operative time or blood loss. This standardized surgical modification represents a feasible preventive strategy to reduce cesarean scar–related morbidity.

**Trial registration:**

ClinicalTrials.gov, NCT07228858. Registered 20 December 2025 (retrospectively registered).

**Supplementary Information:**

The online version contains supplementary material available at 10.1186/s12884-026-09142-w.

## Background

A uterine niche is defined as an indentation of ≥ 2 mm in the myometrium at the site of a cesarean section scar [1]. First described in 2001, niches reflect impaired myometrial healing following cesarean delivery [2]. They are clinically significant because they are associated with abnormal uterine bleeding, postmenstrual spotting, dysmenorrhea, dyspareunia, and chronic pelvic pain [3], and have been linked to secondary infertility and reduced live birth rates after assisted reproductive technologies [4, 5].

Niches also increase the risk of severe complications in subsequent pregnancies, including cesarean scar ectopic pregnancy, placenta accreta spectrum disorders, and uterine rupture [6], with the likelihood of rupture related to niche size and residual myometrial thickness [7]. Reported prevalence rises with the number of cesarean deliveries, reaching up to 61% after a first cesarean, 81% after a second and even higher after a third [8]. Systematic reviews report wide prevalence estimates ranging from 42% [9] to 84% [10], while large niches involving > 50% of the myometrium occur in 11% [11] to 45% of cases [8].

Diagnosis relies primarily on imaging, with saline-infusion sonohysterography considered more reliable than conventional transvaginal sonography [1, 12]. Proposed etiological factors include ischemia and suboptimal uterine closure techniques [13]. Histopathological studies have reported scar endometriosis, endometrial cysts, and cystic adenomyosis, suggesting a possible link between niche formation and iatrogenic endometriosis or adenomyosis [14].

Surgical technique is a key determinant of niche development. Double-layer closure of the uterine incision reduces the risk of niche formation, dehiscence, and rupture compared with single-layer closure [15, 16]. Despite growing evidence on closure techniques, there is limited randomized evidence evaluating preventive scar optimization strategies performed at the time of repeat cesarean delivery. Intraoperative repair of myometrial defects during repeat cesarean delivery has shown promising results in reducing niche formation, supporting the concept that optimizing the scar area at the time of repeat cesarean may enhance myometrial healing [17]. Recently, consensus definitions and diagnostic criteria for cesarean section scar disorder were established using a modified Delphi process [18].

This study aimed to evaluate whether resection of the previous cesarean scar during repeat cesarean delivery using a standardized and technically simple surgical modification can prevent uterine niche formation and associated morbidity without increasing operative risks.

## Methods

### Study design and setting

This was a single-center, prospective, parallel-group, randomized controlled superiority trial with a 1:1 allocation ratio, comparing scar resection versus standard repeat cesarean section for the prevention of uterine niche formation. The study was conducted at the Department of Obstetrics and Gynecology, Benha University Hospital, Benha, Qalyubia, Egypt. Ethical approval was obtained from the Institutional Research Ethics Committee (approval number RC 9-2-2025) prior to participant recruitment. The trial protocol was registered at ClinicalTrials.gov (NCT07228858; retrospectively registered due to administrative delays), and the full protocol is publicly available through the registry. The study protocol, eligibility criteria, and primary outcome were finalized prior to the enrollment of the first participant. Patient recruitment was conducted between February and November 2025.

All participants provided written informed consent before enrolment. Privacy, institutional regulations, and the principles of the Declaration of Helsinki were strictly observed throughout the study. This study is reported in accordance with the CONSORT guidelines for reporting randomized controlled trials. Patients and the public were not involved in the design, conduct, reporting, or dissemination plans of this research. No important changes were made to the trial design, prespecified outcomes, or planned analyses after trial commencement.

### Participants

Multigravida women aged 18–45 years with ≥ 1 prior cesarean delivery at ≥ 28 weeks’ gestation (local fetal viability threshold) presenting to the Department of Obstetrics and Gynecology, Benha University Hospital for repeat cesarean delivery for any indication (elective or urgent) were screened for eligibility. Gestational age was determined according to ACOG Committee Opinion No. 688 [19].

### Inclusion criteria

Participants who provided written informed consent, agreed to 6-month postpartum follow-up and reported no intention to conceive during the follow-up period were eligible.

### Exclusion criteria

Participants were excluded if they had uterine fibroids, multiple gestation, chorioamnionitis, placenta previa, placental abruption, preeclampsia or eclampsia, hepatic or renal dysfunction, uncontrolled diabetes mellitus, antepartum hemoglobin < 10 g/dL, chronic corticosteroid use, smoking, or inability to provide informed consent.

### Randomization and blinding

Eligible participants were randomly assigned in a 1:1 ratio to either the scar-resection group or the control group. A computer-generated permuted-block randomization sequence (block size 10) was prepared by a statistician independent of participant recruitment, intervention delivery, and outcome assessment. Allocation concealment was ensured using sequentially numbered, opaque, sealed envelopes, which were opened by a trained nurse immediately after participant consent and prior to scheduling surgery. Obstetricians performing the cesarean section were informed of group allocation after randomization. Outcome assessors, including the sonographer performing saline-infusion sonohysterography at 6 months postpartum, were blinded to group assignment. Participants were not blinded to group allocation due to the nature of the intervention.

### Intervention

After confirmation of eligibility and informed consent, baseline demographic and clinical variables (age, body mass index (BMI), gestational age, number of prior cesarean sections, being in active labor with cervical dilatation equal to or more than 3 cm, and fetal membranes intact or ruptured) were recorded by the attending staff nurse.

**Fig. 1:**
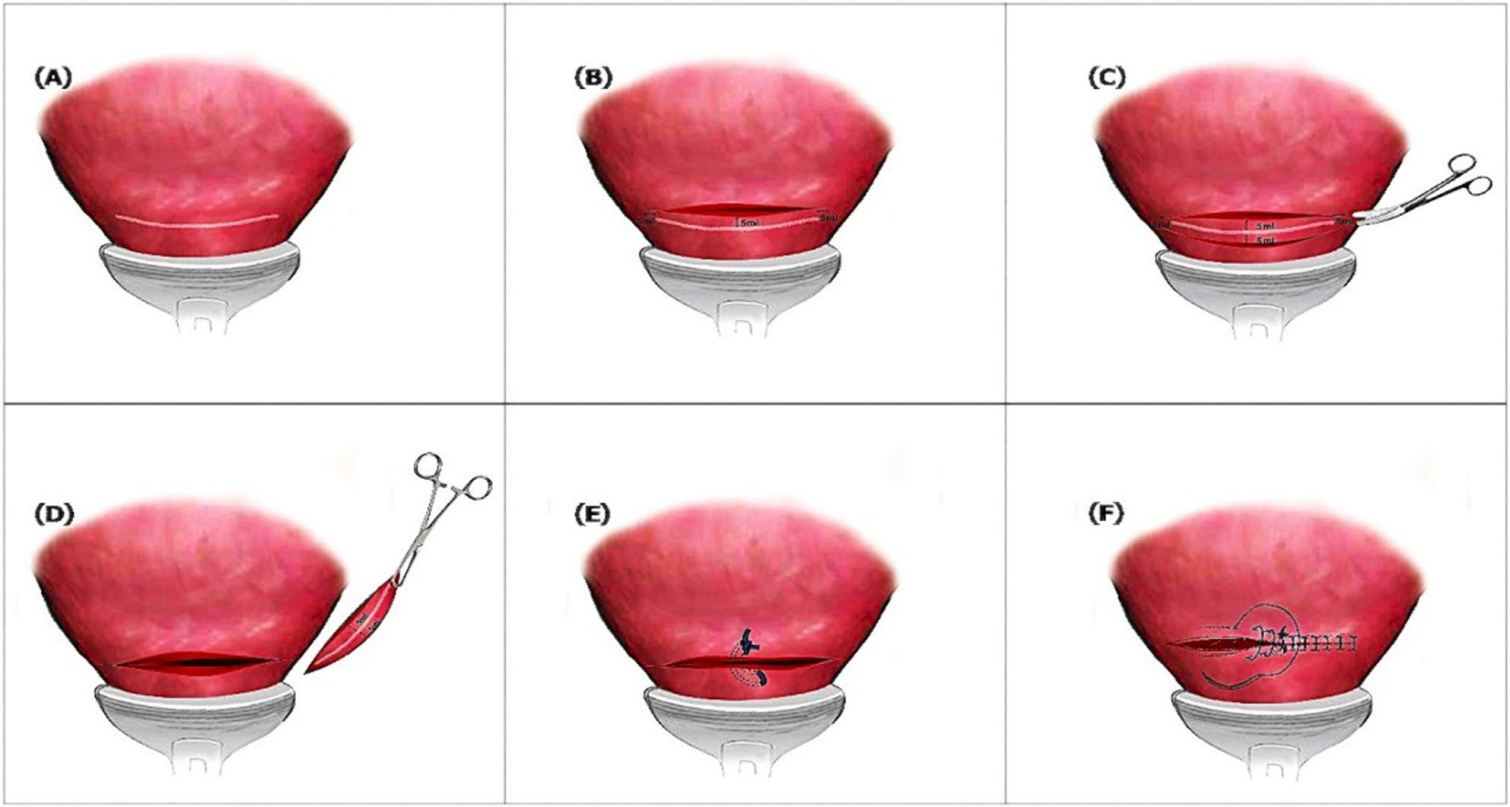
Diagrammatic representation of scar resection technique. **A** Identification of the site of the previous cesarean section/s scar. **B** Incision is made 5 mm cranial to the scar and extended 5 mm on both sides beyond its lateral ends. **C** After fetal delivery, the scar is resected with 5 mm of the myometrium caudal to it. **D** The resulting uterine incision after scar resection. **E** A vertical mattress suture is applied to the center of the uterine incision approximating its cranial and caudal edges. **F** Double layer continuous suturing of the myometrium incorporating and closing the visceral uterine peritoneum with it

Participants were randomly assigned to one of two groups:

- Study group (scar-resection, *n* = 85): During repeat cesarean section, the previous cesarean scar was resected (Fig. 1). The bladder was carefully dissected from the lower uterine segment and then gently retracted caudally to expose the site of the previous cesarean section/s scar. The uterus was incised 5 mm cranial to the scar and extended laterally 5 mm beyond its lateral ends. After fetal delivery, the scar and 5 mm of caudal myometrium were sharply resected. Distances were estimated using sterile surgical calipers to ensure consistency. The resulting uterine incision edges were centralized and approximated with a single vertical mattress suture, followed by double-layer continuous myometrial suturing without locking with closure of the visceral uterine peritoneum incorporated into the second run of myometrial sutures rather than as a separate layer.
- Control group (standard cesarean section, *n* = 85): Standard repeated cesarean section was performed without scar resection, followed by identical double-layer continuous myometrial suturing without locking with closure of the visceral uterine peritoneum incorporated into the second run of myometrial sutures rather than as a separate layer.

All participants received the allocated intervention as planned, and no protocol deviations in intervention delivery were recorded. No modifications to the surgical procedure were made based on labor status or membrane rupture.

### Surgical procedure

**Fig. 2:**
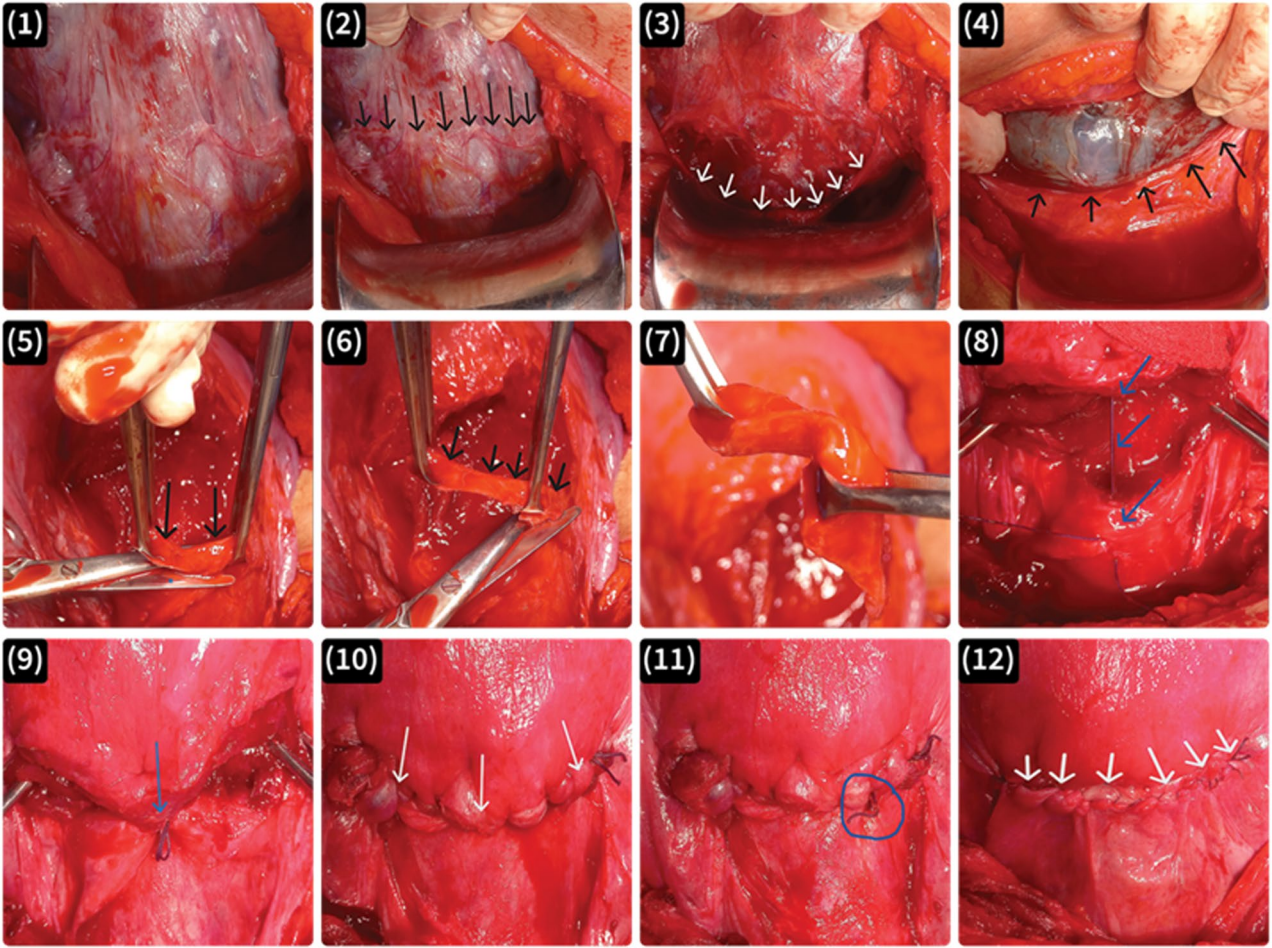
Photographic images of scar resection and uterine incision closure techniques. (**1**) Identification of the previous cesarean scar. (**2**) Black arrows indicate the line of the scar. (**3**) Dissection of the bladder and visceral peritoneal flap (white arrows). (**4**) Incision made 5 mm cranial to the scar and extended 5 mm laterally beyond both ends. (**5**) After fetal delivery, the scar is resected along with 5 mm of caudal myometrium. (**6**) Further resection of the scar to ensure complete removal. (**7**) Scar completely resected. (**8**) A vertical mattress suture applied to the center of the uterine incision, approximating cranial and caudal edges (blue arrows). (**9**) The vertical mattress suture after ligation (blue arrow). (**10**) First layer of continuous, non-locking myometrial suturing. (**11**) Additional hemostatic suture applied to a bleeding site. (**12**) Second layer of continuous, non-locking myometrial suturing incorporating and closing the visceral uterine peritoneum with the second run of myometrial sutures rather than as a separate layer

All procedures were performed at the Department of Obstetrics and Gynecology, Benha University Hospital, by qualified senior obstetricians who were trained in both standard repeated cesarean section and scar resection techniques and performed both interventions in a randomized fashion to avoid operator bias and to ensure consistency across participants. No additional eligibility criteria were applied for the study site or for the clinicians delivering the interventions. Intraoperative hemostasis was ensured, and any additional hemostatic sutures were recorded immediately after the procedure. A synthetic absorbable braided polyglactin 910 (0–1) suture was used for all procedures (Fig. 2).

### Perioperative management

Apart from the allocated interventions, all participants received identical perioperative and postoperative care according to institutional protocols. No additional agents or adjunctive treatments were used in either group. Analgesia and antibiotic prophylaxis were standardized and identical for all participants.

Regarding uterotonic usage;

- Oxytocin (Syntocinon^®^, Novartis Pharma, Egypt): 10 units IV, administered slowly after fetal head delivery as a routine intraoperative uterotonic.
- Carbetocin (Pabal^®^, Draxis/Multipharma, Egypt, under license from Draxis Pharma, Canada): 100 µg IV in 10 ml normal saline, administered as an additional uterotonic if intraoperative uterine atony occurred.
- Methylergometrine (Methergine^®^, Novartis Pharma, Egypt): 200 µg IM every 8 h for the first 24 postoperative hours, as per institutional protocol.
- Misoprostol (Misotac^®^, Sigma Pharmaceutical Industries, Egypt): 400 µg sublingually, administered as an additional uterotonic if postoperative uterine atony occurred.

All additional uterotonics were used only as clinically indicated and was recorded in the participants’ clinical records.

### Outcome assessment

#### Primary outcome

**Fig. 3:**
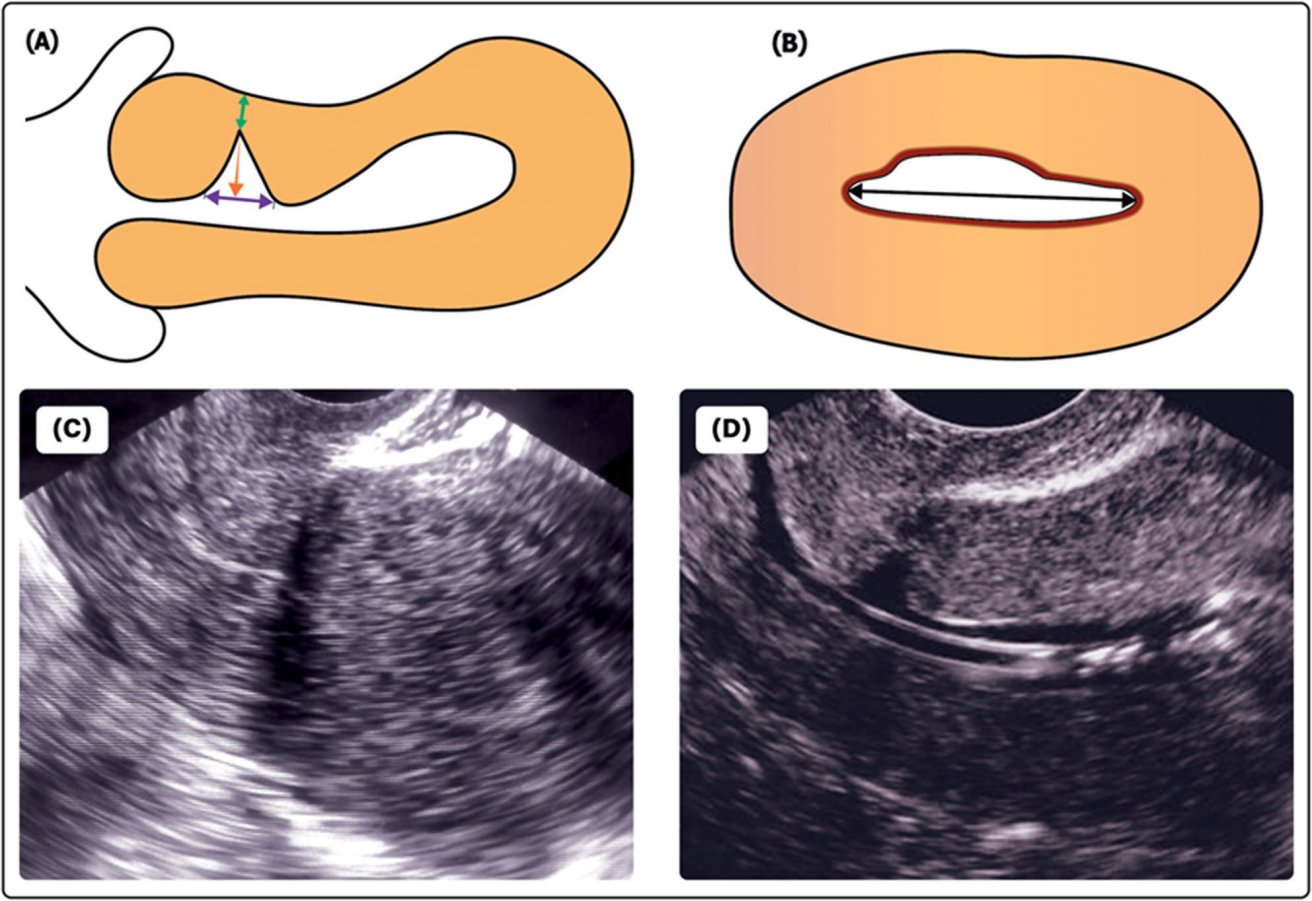
Uterine niche measurements used in the study. **A** Sagittal plane demonstrating niche depth (red arrow), niche length (blue arrow), and residual myometrial thickness (RMT; green arrow). **B** Transverse plane demonstrating niche width (black arrow). **C** Transvaginal ultrasound image of uterine cesarean scar niche. **D** Saline-infusion sonohysterography image of the same case

Incidence of uterine niche formation at 6 months postpartum. Transvaginal ultrasound was first performed to exclude pregnancy or pelvic pathology, followed by saline-infusion sonohysterography (2D, sagittal and transverse views) performed by a blinded sonographer. A niche was defined as ≥ 2 mm indentation in the myometrium at the site of the cesarean section scar. For niche-positive participants, niche depth, length, width, and residual myometrial thickness (RMT) were recorded (Fig. 3).

#### Secondary outcomes

##### Niche measurements at 6 months postpartum

Niche depth, length, width, and RMT were assessed. All measurements were performed by a single experienced, blinded sonographer to ensure consistency (Fig. 3).

##### Total operative time

The operation room nurse recorded the time lapse between the skin incision and the end of skin suturing immediately postoperatively.

##### Total estimated intraoperative blood loss

Blood loss (mL) was calculated as the difference between wet and dry weights of surgical materials (1 g = 1 mL), added to the volume in suction canisters. Suction canister volume was corrected by subtracting irrigation fluid and amniotic fluid as measured intraoperative.

##### The niche-related menstrual cycle bleeding abnormalities at 6 months postpartum

Menstrual cycle bleeding characteristics were assessed as prespecified by the protocol only in niche-positive participants, consistent with the primary hypothesis that menstrual bleeding abnormalities are primarily niche-dependent. Menstrual data were obtained by a blinded obstetrician/gynecologist who was independent of surgical team, documenting number of days of intermenstrual and/or postmenstrual spotting per cycle, number of total bleeding days per cycle, and amenorrhea if present.

### Harms (safety assessment)

Maternal adverse events were prospectively and systematically monitored intraoperative, during the postoperative hospital stay, and until completion of the 6-month postpartum follow-up period, in accordance with the CONSORT extension for reporting harms. Adverse events were not prespecified efficacy outcomes.

Major intraoperative adverse events were defined as excessive blood loss requiring blood transfusion, extension of the uterine incision, bladder or bowel injury, hysterectomy, or any unplanned additional surgical intervention.

Major postoperative adverse events were defined as postpartum hemorrhage requiring blood transfusion or reoperation, endometritis requiring intravenous antibiotic therapy or hospital readmission, surgical site infection requiring procedural intervention, reoperation, admission to the intensive care unit, or maternal death.

Minor postoperative adverse events—including transient postoperative fever, mild postoperative anemia not requiring transfusion (postoperative hemoglobin concentration < 10 g/dL with stable vital signs), superficial wound erythema not requiring medical or surgical intervention, mild gastrointestinal symptoms, and transient urinary symptoms without evidence of structural injury—were recorded and analyzed descriptively but were not classified as major adverse events.

Postoperative pain was evaluated using the Numeric Rating Scale (NRS), graded from 0 (no pain) to 10 (worst pain imaginable). Increased postoperative pain was defined as patient-reported pain requiring additional analgesics beyond the standard institutional post-cesarean analgesic protocol of Diclofenac Potassium 50 mg intramuscularly every 8 h.

### Statistical analysis

All statistical analyses were performed using IBM SPSS Statistics, version 29.0 (IBM Corp., Armonk, NY, USA). A modified intention-to-treat approach including all randomized participants with available primary outcome data was applied. Analyses were conducted on a complete-case basis, with no imputation for missing data, as loss of primary outcome and follow-up was minimal (*n* = 10; 5.9%) and balanced between groups.

Continuous variables were assessed for normality using the Shapiro–Wilk test and visual inspection of histograms. All continuous variables in this study were non-normally distributed and are therefore presented as median and interquartile range (IQR). Categorical variables are presented as frequencies and percentages.

Between-group comparisons of continuous variables—including baseline characteristics, operative outcomes, uterine niche dimensions, residual myometrial thickness, and menstrual parameters—were performed using the Mann–Whitney U test. Categorical variables—including the incidence of uterine niche, active labor, and rupture of membranes—were compared using chi-square tests or Fisher’s exact test, as appropriate.

Effect sizes are reported as Hodges–Lehmann median differences with 95% confidence intervals (CIs) for continuous outcomes, and as relative risk (RR) with 95% CI, absolute risk reduction (ARR), and number needed to treat (NNT) for binary outcomes.

To explore whether the effect of the intervention varied according to the number of previous cesarean sections, effect modification analyses were performed. A multivariable logistic regression model was constructed including study group, number of previous cesarean sections, an interaction term (study group × number of prior cesarean sections), and covariates (active labor, rupture of membranes, BMI, and maternal age). Model calibration was assessed using the Hosmer–Lemeshow goodness-of-fit test, and discrimination was evaluated using the area under the receiver operating characteristic (ROC) curve (AUC). The interaction term significance was assessed and interpreted before reporting stratified or adjusted effects. Additionally, Spearman’s rank correlation coefficients were calculated separately within each study group to assess the association between number of previous cesarean sections and uterine niche formation. This non-parametric correlation was performed due to the non-normal distribution of niche incidence relative to number of prior cesarean sections.

Menstrual outcomes at 6 months postpartum were analyzed only among participants diagnosed with a uterine niche, as prespecified in the protocol. Participants with postpartum amenorrhea were excluded from these analyses.

All statistical tests were two-sided, and a *P* value < 0.05 was considered statistically significant. No adjustments for multiple comparisons were performed, as secondary outcomes were prespecified and considered exploratory. No additional prespecified subgroup analyses or post hoc sensitivity analyses were conducted beyond the planned evaluation of effect modification by number of previous cesarean sections.

### Sample size calculation

Sample size was calculated based on the primary outcome, the incidence of uterine niche formation at 6 months postpartum. Based on prior literature, the incidence in the control group was assumed to be 81% in women with more than 1 prior cesarean section [10]. To detect a 25% relative risk reduction (to 60.75%) in the cesarean scar resection group, with a two-sided α of 0.05 and 80% power, a two-sample comparison of proportions indicated that 76 participants were required per group. Accounting for an anticipated 10% loss to follow-up, 85 women were recruited per group, for a total of 170 participants. No interim analyses were planned or conducted, and no formal stopping guidelines were specified.

## Results

### Participant flow

**Fig. 4:**
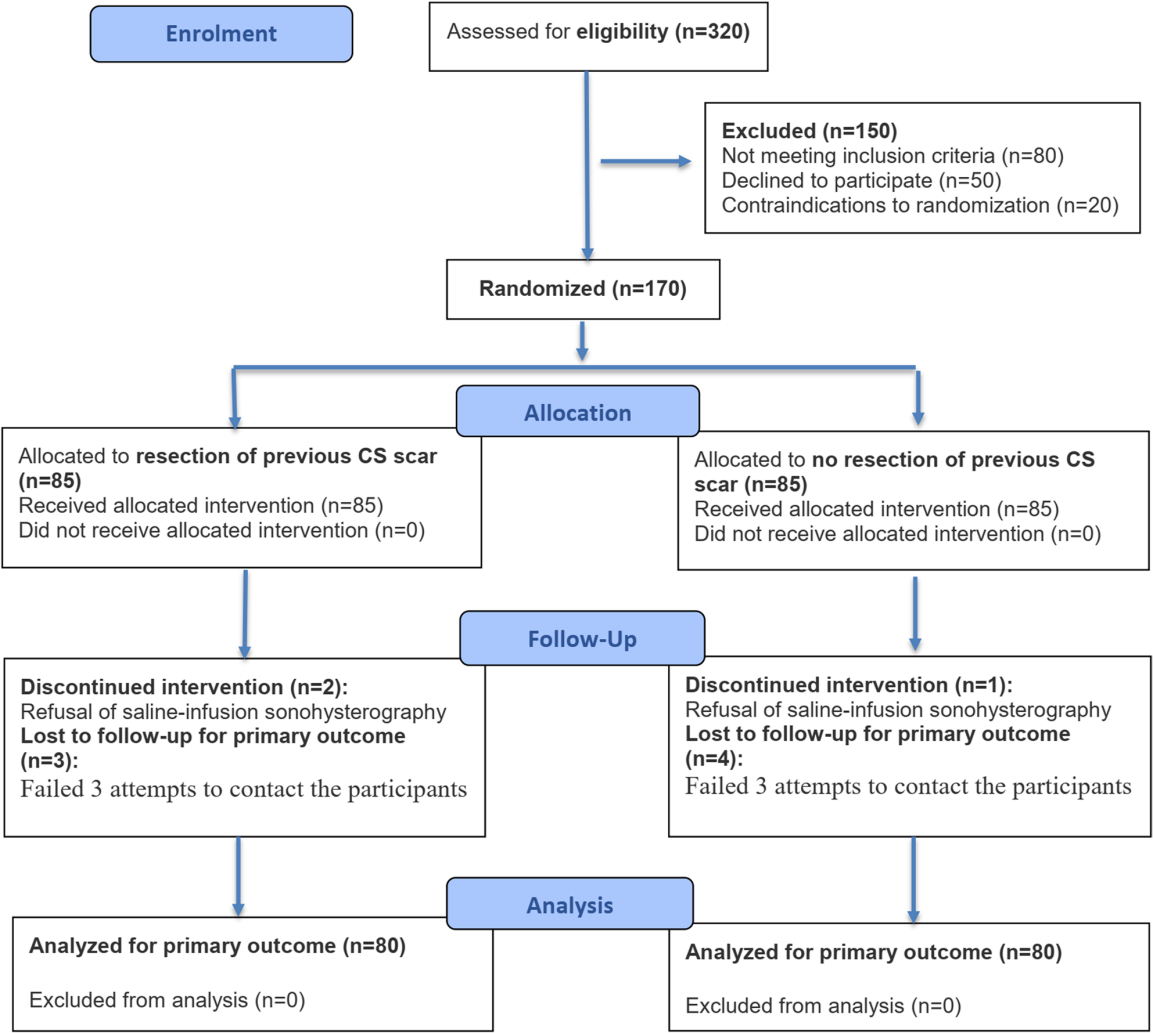
CONSORT flow diagram of participant enrollment, allocation, follow-up, and analysis. Flow diagram showing the progress of participants through the phases of the randomized controlled trial, including enrollment, allocation, follow-up, and analysis

Between February and June 2025, 320 women were screened for eligibility. Of these, 150 were excluded (80 did not meet inclusion criteria, 50 declined participation, 20 had contraindications to randomization), leaving 170 women for randomization. During follow-up, seven participants were lost for follow up, and three declined saline-infusion sonohysterography at 6 months postpartum, resulting in missing primary outcome data for 10 randomized participants (5 in each group). A total of 160 participants (80 per group) completed the 6-month assessment and were included in the primary analysis (modified intention-to-treat). The trial was completed as planned, with no early stopping for efficacy, safety, or feasibility reasons (Fig. 4).

### Baseline demographic and obstetric characteristics

Baseline demographic and obstetric characteristics were similar between the study and control groups (Table 1). No statistically significant differences were observed in age, BMI, gestational age at delivery, number of previous cesarean sections, or preoperative hemoglobin concentration, with medians and interquartile ranges closely matched across groups (*P* = 0.668, 0.851, 0.968, 0.510, and 0.993, respectively).

**Table 1:** Baseline characteristics of study and control groups

Variable	Study group (*n* = 80)	Control group (*n* = 80)	Effect size (95% CI)	*P*-value
Continuous variables				
Age (years)*	32.0 (28.5–36.0)	31.0 (28.0–36.0)	0.0 (–2.0 to 1.0)†	0.668
BMI (kg/m²)*	30.0 (26.5–35.0)	30.0 (26.5–34.5)	0.0 (–2.0 to 2.0)†	0.851
Gestational age (weeks)*	38.0 (37.0–39.0)	38.0 (37.0–39.0)	0.0 (0.0 to 0.0)†	0.968
No. of previous CS*	2.0 (1.0–2.0)	2.0 (1.0–2.0)	0.0 (0.0 to 0.0)†	0.510
Preoperative Hb*	12.35(11.15–13.5)	12.3(11.05–13.45)	0.0 (–0.4 to 0.4)†	0.993
Categorical variables				
Active labor	38 (47.5%)	42 (52.5%)	RR 0.90 (0.57–1.44)	0.528
Rupture of membranes	46 (57.5%)	42 (52.5%)	RR 1.10 (0.72–1.66)	0.526
Controlled diabetes	7 (8.75%)	5 (6.25%)	RR 1.40 (0.47–4.21)	0.878
Elective CS	31 (38.75%)	36 (45%)	RR 0.86 (0.57–1.30)	0.608
Urgent CS	49 (61.25%)	46 (55%)	RR 1.07 (0.76–1.50)	0.711
Emergent CS	0 (0%)	0 (0%)	NA	NA

Data are presented as median (interquartile range) for continuous variables and number (percentage) for categorical variables

*BMI*  body mass index, *CS*  cesarean section, *Hb*  hemoglobin (g/dL), *RR*  relative risk, *CI* confidence interval, *NA*  Not applicable

*P*-values derived from Mann–Whitney U test* for continuous variables and chi-square test for categorical variables

† Hodges–Lehmann median difference

Data regarding the locations, level of emergency and the indications of previous cesarean sections of participants were presented in a supplementary table (Table S1) and they were comparable between the 2 groups with no statistically significant differences.

The proportions of women presenting in active labor (47.5% vs. 52.5%; RR = 0.90, 95% CI 0.57–1.44; *P* = 0.528), with rupture of membranes (57.5% vs. 52.5%; RR = 1.10, 95% CI 0.72–1.66; *P* = 0.526), with controlled diabetes (8.75% vs. 6.25%; RR = 1.40, 95% CI 0.47–4.21; *P* = 0.878), elective CS (38.75% vs. 45%; RR = 0.86, 95% CI 0.57–1.30; *P* = 0.608), urgent CS (61.25% vs. 55%; RR = 1.07, 95% CI 0.76–1.50; *P* = 0.711), and emergent CS (0% vs. 0%) did not differ significantly between groups.

### Operative outcomes

Operative outcomes were comparable between the study and control groups (Table 2). Median total operative time, number of additional hemostatic stitches, estimated intraoperative blood loss, and postoperative hemoglobin concentration did not differ significantly between groups, with values of 42.5 (39.0–47.0) vs. 41.5 (38.5–45.5) minutes, 1.0 (1.0–2.0) vs. 1.0 (0.0–2.0) stitches, 720 (660–780) vs. 715 (635–750) mL, and 11.5 (9.9–12.6) vs. 11.3 (10.05–12.6) g/dL, respectively (*P* = 0.109, 0.726, 0.281, and 0.977).

**Table 2:** Operative outcomes in study and control groups

Variable	Study group (*n* = 80)	Control group (*n* = 80)	Effect size (95% CI)	*P*-value
Total operative time (min)*	42.5 (39.0–47.0)	41.5 (38.5–45.5)	–1.0 (–3.0 to 0.0)†	0.109
Additional stitches required*	1.0 (1.0–2.0)	1.0 (0.0–2.0)	0.0 (0.0 to 0.0)†	0.726
Estimated blood loss (mL)*	720 (660–780)	715 (635–750)	–10 (–30 to 10)†	0.281
Postoperative Hb*	11.5(9.9–12.6)	11.3(10.05–12.6)	0.0 (–0.4 to 0.4)†	0.977
Intraoperative additional uterotonics	6 (7.1%)	7 (8.2%)	RR 0.86 (0.30–2.44)	0.943
Postoperative additional uterotonics	8 (9.4%)	9 (10.6%)	RR 0.89 (0.36–2.21)	0.936

Data are presented as median (interquartile range) for non-normally distributed variables and number (percentage) for categorical variables

*P*-values derived from Mann–Whitney U test* for all non-normally distributed outcomes and chi-square test for categorical variables

*Hb*  hemoglobin (g/dL), *RR*  relative risk, *CI*  confidence interval

† Hodges–Lehmann median difference

Similarly, the proportions of participants requiring additional uterotonics were comparable between groups: intraoperative (7.1% vs. 8.2%; RR = 0.86, 95% CI 0.30–2.44; *P* = 0.943) and postoperative (9.4% vs. 10.6%; RR = 0.89, 95% CI 0.36–2.21; *P* = 0.936).

### Incidence of uterine niche at 6 months postpartum

**Fig. 5:**
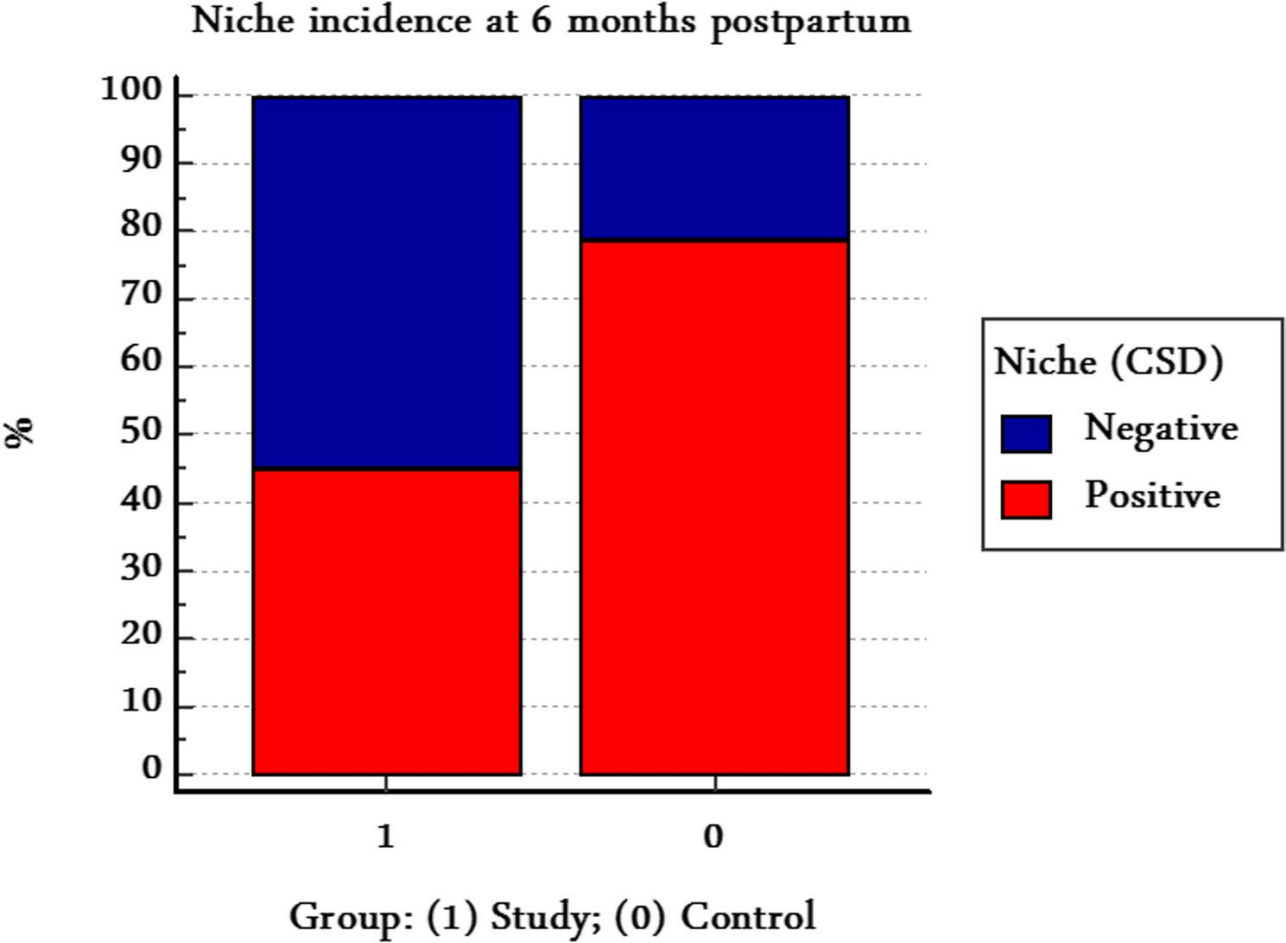
Incidence of uterine niche at 6 months postpartum. The scar-resection group showed a significantly lower incidence of uterine niche compared with the control group (*P*< 0.001)

At 6 months postpartum, saline infusion sonohysterography demonstrated a significantly lower incidence of uterine niche in the scar-resection group compared with the control group (45.0% vs. 78.8%; RR = 0.57, 95% CI 0.44–0.75; *P* < 0.001). The absolute risk reduction (ARR) was 33.8% (95% CI 19.6–47.9), corresponding to a number needed to treat (NNT) of 3, equivalent to 27 fewer niches per 80 women treated. This finding indicates a clinically meaningful reduction in uterine niche formation, achieved without increases in operative time or intraoperative blood loss (Table 3; Fig. 5).

**Table 3:** Incidence of uterine niche at 6 months postpartum

Outcome	Study group (*n* = 80)	Control group (*n* = 80)	Effect size (95% CI)	*P*-value
Niche present, *n* (%)	36 (45.0%)	63 (78.8%)	RR = 0.57 (0.44–0.75); ARR = 33.8% (19.6–47.9); NNT = 3	< 0.001*

Data are presented as number (percentage)

*RR*  relative risk, *ARR*  absolute risk reduction, *NNT*  number needed to treat, *CI*  confidence interval

*P*-value derived from chi-square test

*Significant at *P* < 0.05

### Effect modification by number of previous cesarean sections

To examine whether the effect of scar resection on uterine niche formation varied by number of previous cesarean sections, multivariable logistic regression was performed including study group, number of previous cesarean sections, an interaction term (study group × number of previous cesarean sections), and covariates (active labor, rupture of membranes, BMI, maternal age). Assignment to the control group was independently associated with significantly higher odds of uterine niche formation at 6 months postpartum (adjusted OR 9.11, 95% CI 2.52–32.94; *P* = 0.001) (Table 4).

**Table 4:** Multivariable logistic regression analysis of factors associated with uterine niche formation at 6 months postpartum

Variable	Adjusted OR	95% CI	*P* value
Study group (control vs. scar resection)	9.11	2.52–32.94	0.001
Number of previous cesarean sections	1.45	0.78–2.71	0.240
Active labor (no vs. yes)	1.29	0.62–2.69	0.502
Rupture of membranes (no vs. yes)	1.87	0.91–3.81	0.087
Body mass index (kg/m²)	0.96	0.90–1.02	0.205
Maternal age (years)	1.03	0.95–1.11	0.488

Odds ratios adjusted for all variables included in the model. Uterine niche defined as ≥ 2 mm indentation detected by saline-infusion sonohysterography

In the scar resection group, neither the number of previous cesarean sections nor the interaction term reached statistical significance (Table 5), indicating a consistent protective effect of scar resection across women with differing numbers of previous cesarean deliveries.

**Table 5:** Interaction between study group and number of previous cesarean sections

Variable	Adjusted OR	95% CI	*P* value
Study group (control vs. scar resection)	9.11	2.52–32.94	0.001
Number of previous cesarean sections	1.45	0.78–2.71	0.240
Group × number of previous cesarean sections	1.45	0.78–2.71	0.240

The interaction term assesses whether the effect of the intervention differed according to the number of previous cesarean sections. The non-significant interaction indicates a consistent protective effect of scar resection across increasing cesarean section numbers

**Fig. 6:**
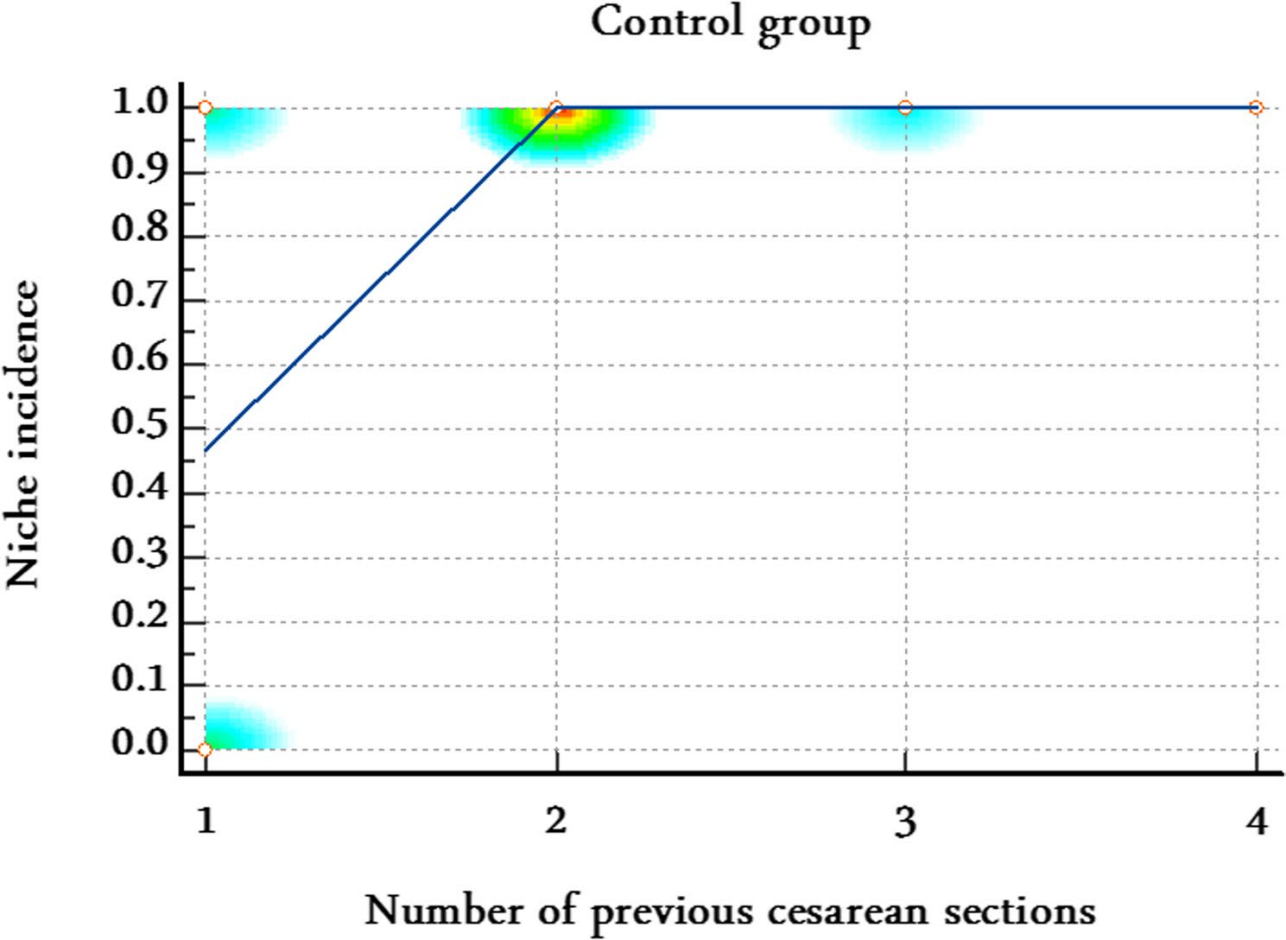
Stratified correlation between number of previous cesarean sections and uterine niche in the control group. The number of previous cesarean sections was strongly and positively correlated with uterine niche occurrence (Spearman’s rho = 0.58, 95% CI 0.42–0.71; *P*< 0.0001)

**Fig. 7:**
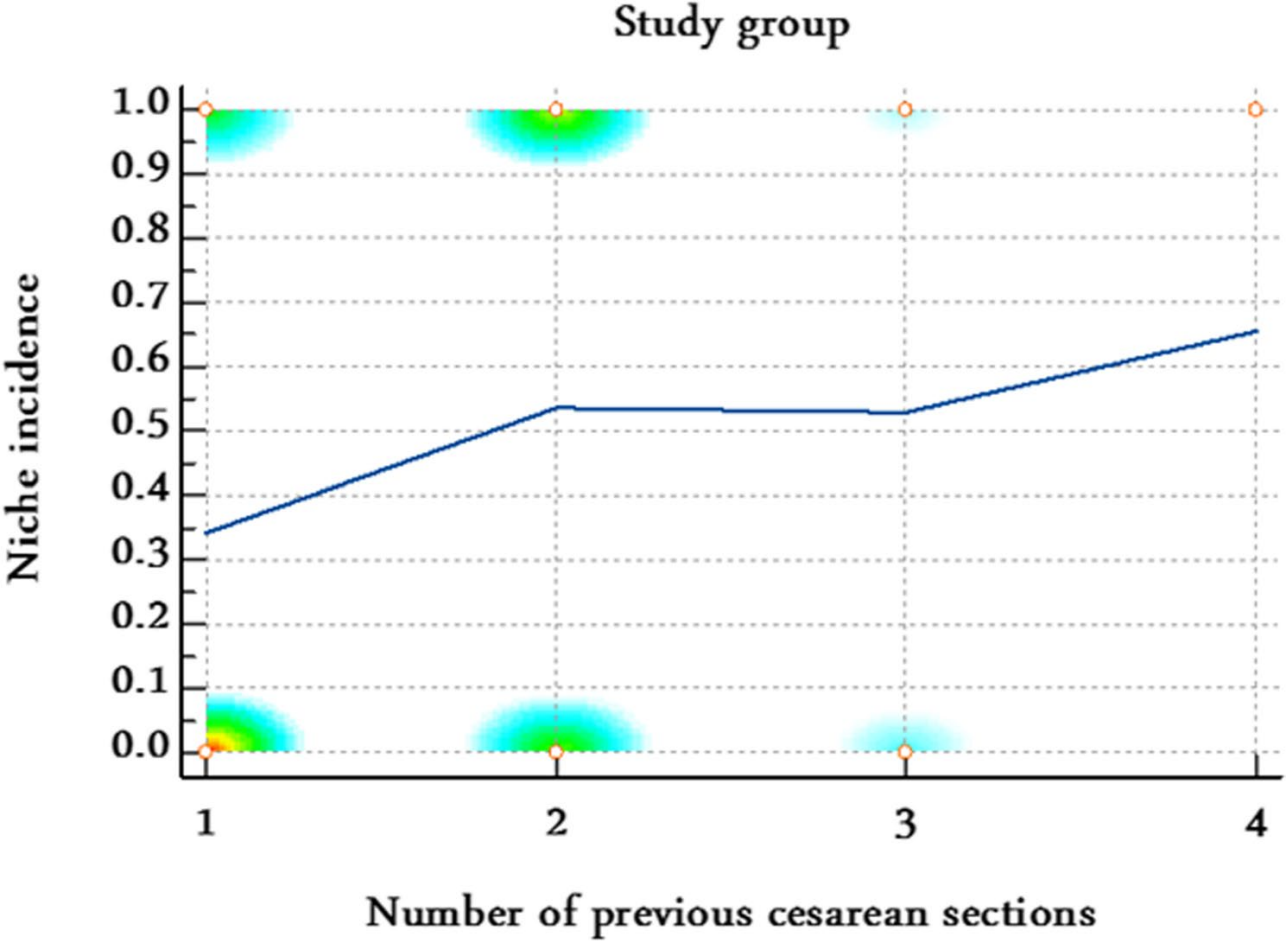
Stratified correlation between number of previous cesarean sections and uterine niche in the scar- resection group. No significant correlation was observed in the scar-resection group (rho = 0.15, 95% CI − 0.07 to 0.36; *P*= 0.18)

Model calibration and discrimination were adequate (Hosmer–Lemeshow *P* = 0.47; AUC = 0.74). Stratified correlation analyses further supported these findings. In the control group, the number of previous cesarean sections was strongly and positively correlated with uterine niche occurrence (Spearman’s rho = 0.58, 95% CI 0.42–0.71; *P* < 0.0001) (Table 6; Fig. 6), while in the scar-resection group, no significant correlation was observed (rho = 0.15, 95% CI − 0.07 to 0.36; *P* = 0.18) (Table 6; Fig. 7) suggesting that scar resection mitigates the cumulative adverse effect of repeated cesarean sections on niche formation.

**Table 6:** Stratified correlation between number of previous cesarean sections and uterine niche occurrence in both groups

Study group	Sample size	Spearman’s rho	95% CI	*P* value
Scar resection group	80	0.15	−0.07 to 0.36	0.18
Control group	80	0.58	0.42–0.71	< 0.0001

Correlation analyses were performed separately for each group to explore the relationship between number of previous cesarean sections and uterine niche formation

### Niche characteristics at 6 months postpartum

Among women who developed a uterine niche, the scar-resection group demonstrated more favorable niche characteristics compared with the control group (Table 7). Median niche depth, length, and width were all significantly smaller in the scar-resection group: 3.0 (3.0–3.5) mm vs. 5.0 (5.0–6.0) mm for depth, 3.0 (2.0–3.0) mm vs. 3.0 (3.0–4.0) mm for length, and 11.0 (11.0–12.0) mm vs. 14.0 (12.0–15.0) mm for width (*P* < 0.0001 for all). Residual myometrial thickness was greater in the scar-resection group, with a median of 8.0 (6.5–8.0) mm compared with 6.0 (4.0–7.0) mm in controls (*P* < 0.0001), corresponding to a Hodges–Lehmann median difference of 2.0 mm (95% CI 1.0–2.0) favoring scar resection.

These results indicate that scar resection produces a smaller, less extensive niche while preserving myometrial integrity, which may have implications for future obstetric outcomes.

**Table 7:** Niche characteristics at 6 months postpartum

Variable	Study group (*n* = 36)	Control group (*n* = 63)	Effect size (95% CI)	*P*-value
Niche depth (mm)*	3.0 (3.0–3.5)	5.0 (5.0–6.0)	2.0 (2.0–2.0)†	< 0.0001
Niche length (mm)*	3.0 (2.0–3.0)	3.0 (3.0–4.0)	1.0 (1.0–1.0)†	< 0.0001
Niche width (mm)*	11.0 (11.0–12.0)	14.0 (12.0–15.0)	2.0 (1.0–3.0)†	< 0.0001
Residual myometrial thickness (mm)*	8.0 (6.5–8.0)	6.0 (4.0–7.0)	–2.0 (–2.0 to − 1.0)†	< 0.0001

Data are presented as median (interquartile range) for non-normally distributed variables. *P*-values derived from Mann–Whitney U test*

†Hodges–Lehmann median difference

#### Cyclic menstrual bleeding characteristics among participants with uterine niche

As prespecified in the protocol, menstrual outcomes were analyzed only among participants diagnosed with a uterine niche. Among 99 participants with a uterine niche (36 in study group, 63 in control), four cases of postpartum amenorrhea (two per group) were excluded, leaving 34 and 61 participants respectively for cyclic menstrual bleeding outcome analysis.

The scar-resection group reported significantly fewer days of postmenstrual and/or intermenstrual spotting; with a median of 2.0 (1.0–3.0) days compared with 5.0 (3.8–5.3) days in the control group (*P* < 0.0001). Total bleeding days per cycle were also shorter in the scar-resection group, with a median of 9.0 (8.0–10.0) days versus 11.0 (10.0–13.3) days in controls (*P* < 0.0001). Hodges–Lehmann median differences indicate clinically meaningful reductions in both bleeding parameters following cesarean scar resection, reflecting functional benefit to patients (Table 8).

**Table 8:** Menstrual characteristics among women with uterine niche

Variable	Study group (*n* = 34)	Control group (*n* = 61)	Effect size (95% CI)	*P*-value
Postmenstrual/intermenstrual spotting (days)*	2.0 (1.0–3.0)	5.0 (3.8–5.3)	2.0 (2.0–3.0)†	< 0.0001
Total bleeding days per cycle*	9.0 (8.0–10.0)	11.0 (10.0–13.3)	3.0 (2.0–3.0)†	< 0.0001

Data are presented as median (interquartile range) for non-normally distributed variables. *P*-values derived from Mann–Whitney U test*

†Hodges–Lehmann median difference

### Harms

No major intraoperative or postoperative adverse events were observed in either the scar-resection group or the control group. Minor postoperative adverse events were observed in both groups and were self-limited or managed conservatively (Table 9). Mild postoperative anemia not requiring transfusion occurred in 22 women (25.9%) in the scar-resection group and 20 women (23.5%) in the control group (*P* = 0.86). Transient postoperative fever was reported in 9 (10.6%) and 8 (9.4%) participants, respectively (*P* = 0.94). Superficial wound erythema occurred in 7 (8.2%) women in the scar-resection group and 6 (7.1%) in the control group (*P* = 0.94). Increased postoperative pain requiring more analgesics beyond the standard hospital protocol was reported by 26 women (30.6%) in the scar-resection group and 24 women (28.2%) in the control group (*P* = 0.85). Mild gastrointestinal symptoms occurred in 6 (7.1%) and 5 (5.9%) participants, respectively (*P* = 0.94), while transient urinary symptoms without evidence of structural injury were reported in 5 (5.9%) women in the scar-resection group and 4 (4.7%) in the control group (*P* = 0.94).

**Table 9:** Maternal minor adverse events following repeat cesarean delivery

Adverse event	Scar resection group (*n* = 85)	Control group (*n* = 85)	*P* value*
Mild postoperative anemia	22 (25.9%)	20 (23.5%)	0.859
Transient postoperative fever	9 (10.6%)	8 (9.4%)	0.936
Superficial wound erythema	7 (8.2%)	6 (7.1%)	0.943
Increased postoperative pain	26 (30.6%)	24 (28.2%)	0.853
Mild gastrointestinal symptoms	6 (7.1%)	5 (5.9%)	0.939
Urinary symptoms	5 (5.9%)	4 (4.7%)	0.940
Any minor adverse event	41 (48.2%)	38 (44.7%)	0.757

Data are presented as number (%). No major intraoperative or postoperative adverse events occurred in either group

*P values were calculated using the chi-square test or Fisher’s exact test, as appropriate

Overall, at least one minor adverse event was recorded in 41 women (48.2%) in the scar-resection group and 38 women (44.7%) in the control group, with no statistically significant difference between groups (*P* = 0.76).

#### Ancillary analyses

No additional, subgroup, or sensitivity analyses were performed beyond those prespecified in the protocol.

## Discussion

This randomized controlled trial demonstrates that resection of the previous cesarean scar during repeat cesarean delivery significantly reduces the incidence and severity of uterine niche, increases residual myometrial thickness, and lessens menstrual morbidity, without prolonging operative time, increasing the need for additional hemostatic sutures, or raising intraoperative blood loss.

The incidence of uterine niche in the control group (78.8%) aligns with previously reported prevalence estimates, which vary widely depending on diagnostic criteria, imaging modality, and the number of prior cesarean deliveries [1, 3, 6, 8]. In contrast, the scar-resection group exhibited a substantially lower incidence (45%), corresponding to a statistically significant reduction in risk (RR = 0.57, 95% CI 0.44–0.75; *P* < 0.001). The absolute risk reduction of 33.8% and a number needed to treat of 3 indicate that this difference is statistically significant and clinically meaningful. These findings support the hypothesis that the presence of unresected, poorly vascularized fibrotic tissue at the previous cesarean site impairs myometrial healing and contribute to niche formation [13].

Corrective procedures, such as laparoscopic or hysteroscopic niche resection [4, 20–22], in women with symptomatic uterine niches—where the scar tissue is surgically excised and healthy myometrium is restored—have been shown to improve abnormal bleeding, restore myometrial integrity, and increase residual myometrial thickness. These findings provide strong biological plausibility for the preventive scar resection hypothesis.

In particular, Vissers et al. [20] demonstrated sustained improvement in reproductive outcomes, reduction in niche volume, and increased residual myometrial thickness following laparoscopic niche resection, reinforcing the concept that surgical optimization of the cesarean scar can yield lasting structural and clinical benefits.

Additionally, Siraj et al. [17] described a modified uterine closure technique during repeat cesarean delivery, involving mobilization and precise apposition of retracted myometrial edges at the scar site, which achieved a mean residual myometrial thickness of 8.4 mm at three months postpartum without prolonging operative time or increasing blood loss.

Although Siraj et al. study was non-randomized and limited to relatively small number of cases with incidentally detected large uterine myometrial defects at the previous cesarean scar site during performing repeated CS; it supports the principle that intraoperative scar optimization enhances myometrial integrity.

Our findings extend this concept by demonstrating, in a randomized controlled setting, that preventive scar resection during repeat cesarean delivery significantly reduces both the incidence and severity of uterine niche formation and associated menstrual morbidity, potentially obviating the need for subsequent corrective procedures.

Larger niche dimensions and reduced residual myometrial thickness have been strongly associated with abnormal uterine bleeding and pelvic pain [3, 18, 23]. Accordingly, the significantly smaller niche dimensions and greater residual myometrial thickness observed in the scar-resection group provide a plausible mechanistic explanation for the reduction in postmenstrual/intermenstrual bleeding and total bleeding days per cycle.

We deliberately excluded participants without a uterine niche from the assessment of cyclic menstrual bleeding, as including them could have diluted clinically relevant differences and reduced interpretability. This approach is particularly important because mild bleeding abnormalities may occur in the early postpartum cycles even in the absence of an anatomical defect. Our findings, therefore, specifically reflect the impact of uterine niche on menstrual outcomes. These results are consistent with a recent meta-analysis demonstrating that cesarean scar defects significantly increase the risk of abnormal uterine bleeding [24].

Regarding the timing of cesarean scar niche evaluation, Jiang et al. [25] compared ultrasonic assessments of cesarean scar defects at multiple postpartum time points and found that the size of the scar defect remained stable between 6 months and 1 year after cesarean section. Therefore, we believe that evaluating cesarean scar defects and their associated symptoms at 6 months, as performed in our study, provides a valid and reliable assessment.

### Interpretation and safety

By excising fibrotic scar tissue and re-approximating healthy myometrium, scar resection during repeat cesarean delivery likely enhances uterine healing and reduces niche development. In this study, the procedure was not associated with an increase in major intraoperative or postoperative adverse events. No cases of excessive hemorrhage requiring transfusion, extension of the uterine incision, visceral injury, hysterectomy, or other major complications were observed in either group.

Minor postoperative adverse events—including transient fever, mild anemia not requiring transfusion, increased postoperative pain, superficial wound erythema, mild gastrointestinal symptoms, and transient urinary complaints—occurred at comparable frequencies in both groups and were self-limited or managed conservatively.

Although the study was not powered to detect rare complications, the observed safety profile suggests that this standardized surgical modification can be performed without an apparent increase in short-term maternal morbidity in carefully selected patients, supporting its feasibility for incorporation into routine clinical practice.

### Clinical implications

Scar resection during repeat cesarean delivery appears to be a feasible and safe surgical modification that may reduce the incidence and severity of uterine niche. The associated increase in residual myometrial thickness and reduction in niche-related bleeding suggest improved uterine scar integrity. No additional operative risks were observed, indicating that the procedure can be performed without obvious short-term maternal harm. If these findings are confirmed in larger multicenter studies, preventive scar resection may reduce abnormal uterine bleeding, decrease the need for secondary niche repair, and potentially improve reproductive outcomes.

### Strengths and limitations

The strengths of this study include its randomized design, standardized surgical technique, blinded outcome assessment, and the use of saline-infusion sonohysterography, which offers greater sensitivity for niche detection than conventional ultrasonography [1, 12].

Limitations include the single-center design and the exclusion of women with major obstetric or medical comorbidities. These criteria were applied to reduce confounding and operative risk that may limit generalizability to broader or higher-risk populations.

Additionally, the study was not powered to detect rare surgical complications, as safety outcomes were not prespecified efficacy endpoints. Therefore, while no major adverse events were observed, the possibility of uncommon complications cannot be excluded.

### Future research

Further large multicenter randomized trials are needed to confirm the efficacy and safety of preventive scar resection during repeat cesarean delivery.

Long-term follow-up studies should evaluate fertility outcomes, subsequent pregnancy complications, and risks such as placenta accreta spectrum and uterine rupture.

Comparative studies of preventive scar resection versus secondary niche repair would help clarify relative benefits, risks, and cost-effectiveness.

Further comparative studies of postoperative inflammatory response, including changes in C-reactive protein and total and differential leukocyte counts should be carried out to asses for the impact of the intervention technique on them.

In addition, molecular and histopathological investigations including examinations of the resected cesarean scars may provide insights into the mechanisms underlying improved uterine healing.

## Conclusion

Resection of the previous cesarean scar during repeat cesarean delivery was associated with a reduced incidence and severity of uterine niche, increased residual myometrial thickness, and decreased niche-related bleeding, without apparent increases in operative time or intraoperative blood loss.

This standardized surgical modification may help reduce long-term gynecologic morbidity related to cesarean scar defects. Confirmation in larger multicenter randomized trials with extended follow-up is needed to evaluate potential effects on fertility, subsequent pregnancy outcomes, and obstetric complications.

## Supplementary Information

Supplementary Material 1. Table S1. Locations, level of emergency and indications of previous CS of participants.

## Data Availability

The datasets used and/or analyzed during the current study are available from the corresponding author upon reasonable request.

## Abbreviations

CS: cesarean section
RMT: residual myometrial thickness
PMS/IMS: postmenstrual/intermenstrual spotting
TBD: total bleeding days
2D: two dimensional
IQR: interquartile range
CI: confidence interval
ARR: absolute risk reduction
CSD: cesarean scar defect
AUC: area under the receiver operating characteristic (ROC) curve
