# Supplementary material for: Uterine cesarean scar resection during repeat cesarean delivery to prevent uterine niche formation: a randomized controlled trial
Source: BMC Pregnancy Childbirth. 2026 May 4;26:489. doi: 10.1186/s12884-026-09142-w (PMC13141410; doi:10.1186/s12884-026-09142-w)
Supplement: Supplementary file 1 — Supplementary Material 1. Table S1. Locations, level of emergency and indications of previous CS of participants. [file 12884_2026_9142_MOESM1_ESM.docx]

**Table S1. Locations, level of emergency and indications of previous CS of participants**

| **Variable** | **Pre CS in Study group (n=138)** | **Pre CS in Control group (n=143)** | **P-value** |
| --- | --- | --- | --- |
| **Location** |  |  |  |
| BUH | 72(52.17%) | 78(54.55%) | 0.771 |
| MH | 41(29.71%) | 38(26.57%) | 0.758 |
| Private sector | 25(18.12%) | 27(18.88%) | 0.944 |
| **Level of emergency** |  |  |  |
| Elective CS | 63(45.65%) | 69(48.25%) | 0.766 |
| Urgent CS | 68(49.28%) | 66(46.16%) | 0.719 |
| Emergent CS | 7(5.07%) | 8(5.59%) | 0.966 |
| **Indication of CS** |  |  |  |
| CPD | 52(37.68%) | 55(38.46%) | 0.934 |
| Failure to progress | 20(14.49%) | 16(11.19%) | 0.773 |
| Fetal distress | 12(8.70%) | 14(9.79%) | 0.925 |
| Fetal macrosomia | 4(2.90%) | 5(3.50%) | 0.962 |
| Fetal malpresentation | 20(14.49%) | 21(14.69%) | 0.986 |
| Cord presentation | 1(0.72%) | 1(0.70%) | 0.999 |
| Placenta previa | 3(2.17%) | 4(2.79%) | 0.962 |
| Accidental hemorrhage | 1(0.72%) | 2(1.40%) | 0.966 |
| Sever PE/ Eclampsia | 8(5.70%) | 7(4.89%) | 0.946 |
| Postdate pregnancy | 5(3.62%) | 3(2.10%) | 0.910 |
| ≥Previous 2CS | 12(8.70%) | 15(10.49%) | 0.878 |

*The numbers presented were the total number of previous CS performed in all participants in each group. Data are presented as number (%).*

**P values were calculated using the chi-square test or Fisher’s exact test, as appropriate.
Pre = previous; CS = cesarean section; BUH = Benha University Hospital; MH = other maternity hospitals; CBD = cephalo pelvic disproportion; PE= preeclampsia.*
